# Viscossuplementação com ácido hialurônico de alto peso molecular para tratamento da osteoartrite do quadril: Revisão sistemática e metanálise atualizadas de estudos clínicos randomizados controlados

**DOI:** 10.1055/s-0046-1819580

**Published:** 2026-04-22

**Authors:** Tales Pasqualotto, Eric Pasqualotto, Leonardo Salvatore Migliardi, Laura Lunelli, Letícia Oliveira Lunelli, Renan Vinicius Romano Martinelli

**Affiliations:** 1 Departamento de Medicina, Universidade Alto Vale do Rio do Peixe, Caçador, SC, Brasil; 2 Departamento de Cirurgia, Universidade Federal de Santa Catarina, Florianópolis, SC, Brasil; 3 Departamento de Ortopedia e Traumatologia, Hospital Governador Celso Ramos, Florianópolis, SC, Brasil; 4 Departamento de Medicina, Universidade do Sul de Santa Catarina, Palhoça, SC, Brasil; 5 Departamento de Ortopedia e Traumatologia, Hospital Municipal do Tatuapé, São Paulo, SP, Brasil; 6 Departamento de Ortopedia e Traumatologia, Hospital Regional de São José Dr. Homero de Miranda Gomes, São José, SC, Brasil

**Keywords:** ácido hialurônico, osteoartrite, quadril, hip, hyaluronic acid, osteoarthritis

## Abstract

**Objetivo:**

Avaliar a eficácia e a segurança do ácido hialurônico de alto peso molecular (AHAPM) em comparação a outros métodos para o tratamento da osteoartrite (OA) do quadril

**Métodos:**

Esta revisão sistemática e metanálise seguiu as diretrizes Preferred Reporting Items for Systematic Reviews and Meta-Analyses (PRISMA). Foram incluídos estudos clínicos randomizados (ECRs) que compararam o AHAPM com outras terapias (corticosteroides, plasma rico em plaquetas, solução salina ou ácido hialurônico de baixo peso molecular) para o tratamento da OA do quadril. As diferenças médias (DMs) ou diferenças médias padronizadas (DMPs) de desfechos contínuos foram calculadas com intervalos de confiança (IC) de 95%.

**Resultados:**

Quatro ECRs foram incluídos, com um total de 823 pacientes com OA do quadril, dos quais 408 (49,5%) foram tratados com AHAPM. A idade média dos pacientes foi de 60,1 (±10,21) anos. Não foram observadas diferenças significativas entre os grupos em relação à dor (DMP, −0,30 pontos; índice de confiança [IC] 95%, −1,60–0,99), índice de Lequesne (DM, 1,30 pontos; IC 95%, −8,83–11,44), pontuação total do Western Ontario and McMaster Universities Osteoarthritis Index (WOMAC) (DM, −9,26 pontos; IC 95%, −51,33–32,56), rigidez segundo o WOMAC (DM, −0,93 pontos; IC 95%, −12,30–10,45), função física segundo o WOMAC (DM, −0,15 pontos; IC 95%, −7,24–7,60) e autoavaliação global pelo paciente (DM, −1,95 pontos; IC 95%, −27,49–23,59).

**Conclusão:**

Não foram observadas diferenças significativas entre o AHAPM e outros tratamentos em relação ao alívio da dor e à recuperação funcional em pacientes com OA do quadril. No entanto, mais estudos clínicos randomizados de alta qualidade são necessários para avaliação do AHAPM no tratamento da OA do quadril.

## Abstract (en)

**Objective:**

To evaluate the efficacy and safety of high molecular weight hyaluronic acid (HMWHA) versus other therapies for hip osteoarthritis (OA) management.

**Methods:**

The present systematic review and meta-analysis followed the Preferred Reporting Items for Systematic Reviews and Meta-Analyses (PRISMA) guidelines. Randomized controlled trials (RCTs) comparing HMWHA versus other therapies (corticosteroids, platelet-rich plasma, saline, or low molecular weight hyaluronic acid) for hip OA treatment were included. Mean differences (MDs) or standardized mean differences (SMDs) were calculated for continuous outcomes with 95% confidence intervals (CIs).

**Results:**

Four RCTs were included, involving 823 patients with hip OA, of whom 408 (49.5%) were treated with HMWHA. The mean age of the patients was 60.1 (±10.21) years. No significant differences were observed between groups for pain (SMD −0.30 points; 95% CI −1.60 to 0.99), Lequesne index (MD 1.30 points; 95% CI −8.83 to 11.44), WOMAC total (MD −9.26 points; 95% CI −51.33 to 32.56), WOMAC stiffness (MD −0.93 points; 95% CI −12.30 to 10.45), WOMAC physical function (MD −0.15 points; 95% CI −7.24 to 7.60), and patient global self-assessment (MD −1.95 points; 95% CI −27.49 to 23.59).

**Conclusion:**

No significant differences were observed between HMWHA and other treatments regarding pain relief and functional recovery in patients with hip OA. However, further high-quality RCTs are needed to evaluate the HMWHA in the treatment of hip OA.

## Introdução

A osteoartrite (OA) do quadril é uma doença articular crônica, degenerativa e multifatorial, que acomete cerca de 6,4% da população mundial e é uma das principais causas de dor e incapacidade. 1 2 É caracterizada por deterioração progressiva da cartilagem e alterações estruturais nas articulações, decorrentes de mecanismos mecânicos e inflamatórios. 3 Apesar da existência de diversos tratamentos não cirúrgicos, muitos pacientes acabam apresentando declínio funcional substancial, tornando a artroplastia do quadril a opção terapêutica definitiva. 1

Diversas abordagens não cirúrgicas são recomendadas para o manejo da OA do quadril, incluindo estratégias não farmacológicas, como exercícios, controle de peso e fisioterapia, 4 bem como analgésicos e anti-inflamatórios não esteroides (AINEs), que continuam sendo tratamentos de primeira linha apesar dos riscos associados. 1 4 Injeções intra-articulares de corticosteroides e ácido hialurônico (HA) também são utilizadas, embora sua eficácia na redução da dor seja variável. 4 5 A viscossuplementação com HA tem recebido crescente atenção, particularmente com formulações de alto peso molecular, como Hylan G-F 20, cuja maior elasticidade e viscosidade mimetizam bem as propriedades do fluido sinovial nativo. 1 6

A viscossuplementação com HA de alto peso molecular (HAAPM) tem sido investigada como opção no tratamento da OA do quadril, com estudos relatando melhora em dor e função. 5 6 No entanto, os achados ainda são inconsistentes em relação à abordagem ideal para o tratamento da OA do quadril, em grande parte devido à heterogeneidade metodológica entre os estudos. Portanto, essa revisão sistemática e metanálise visa atualizar e sintetizar as evidências de estudos clínicos randomizados (ECRs) sobre a eficácia e segurança do AHAPM no tratamento da OA do quadril.

## Métodos

Esta revisão sistemática seguiu as orientações Preferred Reporting Items for Systematic Reviews and Meta-Analysis (PRISMA). 7 O protocolo do estudo foi registrado no International Prospective Register of Systematic Reviews (PROSPERO) 8 sob o número CRD420251123450.

### Estratégia de Busca e Extração de Dados

Buscas sistemáticas foram realizadas nas bases de dados PubMed, Embase e Cochrane Library, desde sua criação até janeiro de 2025, utilizando a seguinte estratégia de busca: ( *Hylan G-F 20* OU *high molecular weight hyaluronic acid* [em português, ácido hialurônico de alto peso molecular] OU *HMWHA* [em português, HAAPM] E ( *hip osteoarthritis* [em português, osteoartrite do quadril OU *hip OA* [em português, OA do quadril] OU HOA). As referências dos artigos incluídos e das revisões sistemáticas da literatura foram avaliadas visando a inclusão de mais estudos. Dois autores (TP e EP) extraíram, de forma independente, as características basais e os desfechos dos artigos de acordo com os critérios de busca predefinidos. Três autores (TP, EP e RVRM) resolveram as divergências por consenso.

### Critérios de Elegibilidade

Foram incluídos estudos que atendiam aos seguintes critérios: (1) ECRs; (2) comparação entre AHAPM e outras modalidades terapêuticas (corticosteroides, plasma rico em plaquetas [PRP], solução salina ou ácido hialurônico de baixo peso molecular [AHBPM]); (3) pacientes com OA do quadril; e (4) relato de pelo menos um dos desfechos de interesse. Estudos que seguiram os seguintes critérios foram excluídos (1) estudos não randomizados; (2) populações não relevantes; e (3) intervenções não relevantes.

### Desfechos e Definições

Os desfechos de interesse foram: (1) dor, (2) índice de Lequesne, (3) pontuação total do Western Ontario and McMaster Universities Osteoarthritis Index (WOMAC), (4) rigidez segundo o WOMAC, (5) função física segundo o WOMAC e (6) autoavaliação global pelo paciente.

O desfecho de dor geral foi avaliado usando a principal medida de dor em cada estudo (Escala Visual Analógica [EVA] ou WOMAC para dor, nos quais pontuações mais altas indicam piora).

O WOMAC é um questionário amplamente utilizado, desenvolvido para avaliação de dor, rigidez articular e função física em indivíduos com OA. As pontuações variam de 0 a 96, sendo que valores mais altos refletem maior incapacidade.

### Avaliação do Risco de Viés

A ferramenta Cochrane Collaboration para avaliação do risco de viés em ensaios randomizados (Rob-2) foi utilizada para avaliar a qualidade de cada ECR. 9 Cada ensaio recebeu uma pontuação de baixo risco, alguma preocupação ou alto risco de viés em cinco domínios: processo de randomização, desvios das intervenções planejadas, ausência de dados sobre os desfechos, mensuração do desfecho e seleção dos resultados relatados. Dois autores independentes (TP e EP) realizaram a avaliação do risco de viés, e quaisquer divergências foram resolvidas por consenso com o autor sênior. O viés de publicação foi analisado por meio da inspeção visual de gráficos de funil. Nenhuma análise quantitativa foi realizada devido ao pequeno número de estudos incluídos (n < 10). 10

### Análise Estatística

Os efeitos do tratamento de desfechos contínuos foram comparados usando diferenças médias (DM) ou diferenças médias padronizadas (DMP) com intervalos de confiança (ICs) de 95%. A heterogeneidade foi avaliada com o teste Q de Cochran e I ^2^ ; valores de *p* < 0,10 e I ^2^ > 25% foram considerados indicativos de heterogeneidade significativa. 11 Modelos de efeitos aleatórios de máxima verossimilhança restrita ( *restricted maximum likelihood* , REML, em inglês) foram usados em todos os desfechos. 12 Para aumentar a robustez da inferência, os ICs de 95% foram ajustados utilizando o método de Hartung-Knapp. Análises de sensibilidade do tipo *leave-one-out* identificaram estudos influentes e seu efeito nas estimativas agrupadas dos desfechos de dor. O tratamento e a conversão dos dados seguiram as diretrizes do *Cochrane Handbook for Systematic Reviews of Interventions* . 13 As análises estatísticas foram realizadas com o *software* estatístico R (R Foundation for Statistical Computing), versão 4.4.1.

## Resultados

### Seleção e Características dos Estudos

**Fig. 1:**
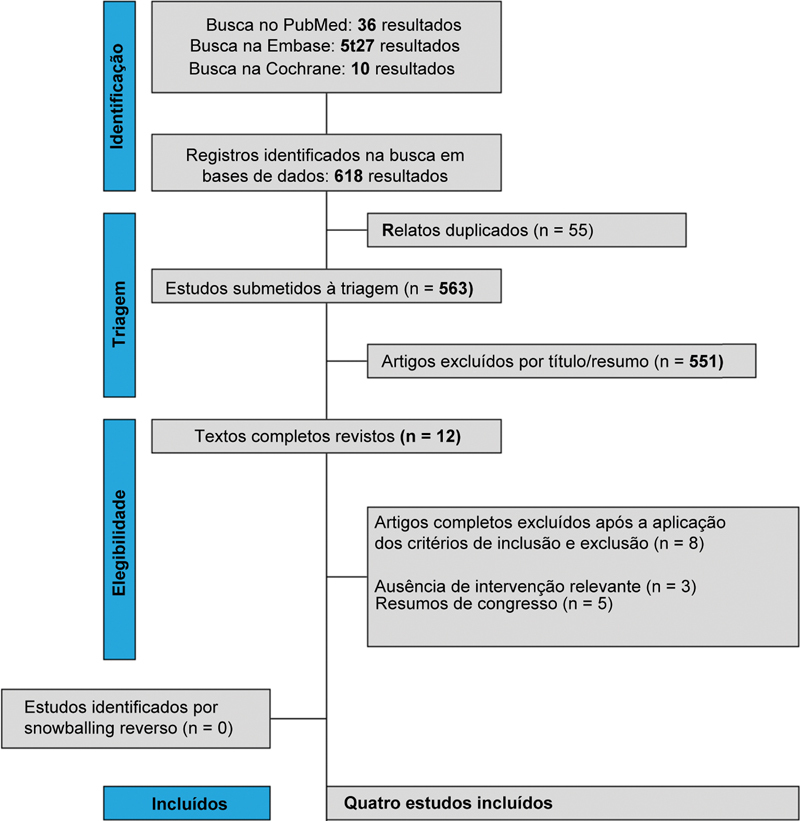
Fluxograma segundo os Preferred Reporting Items for Systematic Review and Meta-Analysis (PRISMA) de triagem e seleção dos estudos.

A busca inicial identificou 618 registros, como ilustrado na Fig. 1 . Após a remoção de duplicatas e a triagem dos estudos por título e resumo, 12 artigos completos atenderam aos critérios para avaliação detalhada. Dentre esses, 4 ECRs foram selecionados para inclusão, abrangendo um total de 823 pacientes com OA do quadril, dos quais 408 (49,5%) receberam AHAPM. 1 5 6 14 O período de acompanhamento variou de 6 meses a 26 semanas. A idade média dos pacientes foi de 60,1 anos, sendo 57,5% do sexo feminino. As características detalhadas dos estudos e dos participantes são apresentadas na Tabela 1 .

**Tabela 1:** Características dos estudos e participantes incluídos na metanálises

Estudo	País	Intervenção	Controle	Acompanhamento	Tamanho da amostra, GI/GC, n	Idade(anos), GI/GC, média ± DP	Sexo (M:F), GI/GC, n	IMC (kg/m ^2^ ) GI/GC, média ± DP	EVA pré-tratamento (GI/GC), média ± DP	Índice de Lequesne pré-tratamento, média ± DP
Spitzer 1 (2010)	EUA	Duas injeções IA de 2 mL de Hylan G-F 20 (administradas com intervalo de 2 semanas)	Uma injeção IA de 2 mL de AMP (40 mg) seguida de uma injeção simulada 2 semanas depois	26 semanas	156/156	59 ± 12/59 ± 11	75:81/76:80	29,3 ± 5,5/29,4 ± 6,0	NA	NA
Tikiz 6 (2005)	Turquia	Hylan G-F 20 (SynviSC, 2,0 mL)	Solução de AHBPM (Ostenil, 2,0 mL)	6 meses	18/25	60,4 ± 9,6/58,8 ± 9,8	4:14/5:20	29,8 ± 3,9/ 28,7 ± 4,3	6,7 ± 1,7/7,2 ± 1,5	11,8 ± 3,3/11,4 ± 4,6
Brander 5 (2019)	Canadá	Hylan G-F 20, uma injeção IA de 6 mL	Salina tamponada com fosfato (uma injeção IA de 6 mL)	26 semanas	182/175	60,8 ± 10,0/59,8 ± 8,8	76:106/70:105	30,9 ± 14,2/29,1 ± 7,6	NA	NA
Nouri 14 (2022)	Irã	Injeção de 2,5 mL com 50 mg de AHAPM como fonte de fermentação linear	5 mL de PRP autólogo	6 meses	29/32	60,93 ± 4,54/58,22 ± 5,10	22:07/22:10	27,62 ± 2,25/27,72 ± 2,11	8,10 1,18/7,63 1,31	12,52 ± 2,34/12,20 ± 2,18

**Abreviaturas** : AHBPM, ácido hialurônico de baixo peso molecular; AHAPM, ácido hialurônico de alto peso molecular; AMP, acetato de metilprednisolona; DP, desvio padrão; EUA, Estados Unidos da América; GC, grupo controle; GI, grupo de intervenção; IA, intra-articular; IMC, índice de massa corporal; NA, não disponível; PRP, plasma rico em plaquetas.

### Análise Agrupada de Todos os Estudos

**Fig. 2:**
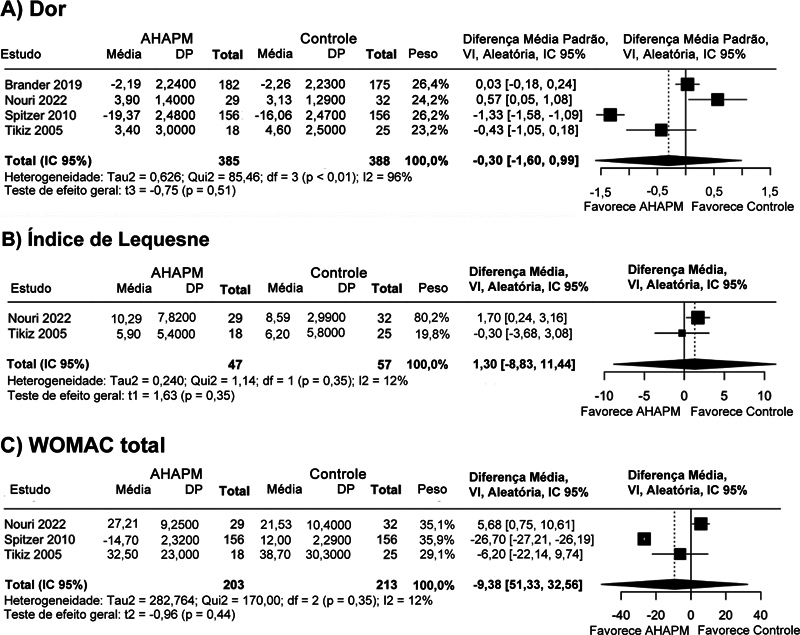
Gráficos de floresta comparando o ácido hialurônico de alto peso molecular (AHAPM) e outras terapias. ( **A** ) Dor. ( **B** ) Índice de Lequesne. ( **C** ) Pontuação total do Western Ontario and McMaster Universities Osteoarthritis Index (WOMAC). DP, Desvio-padrão; df, graus de liberdade; IC, intervalo de confiança; VI, variância inversa.

**Fig. 3:**
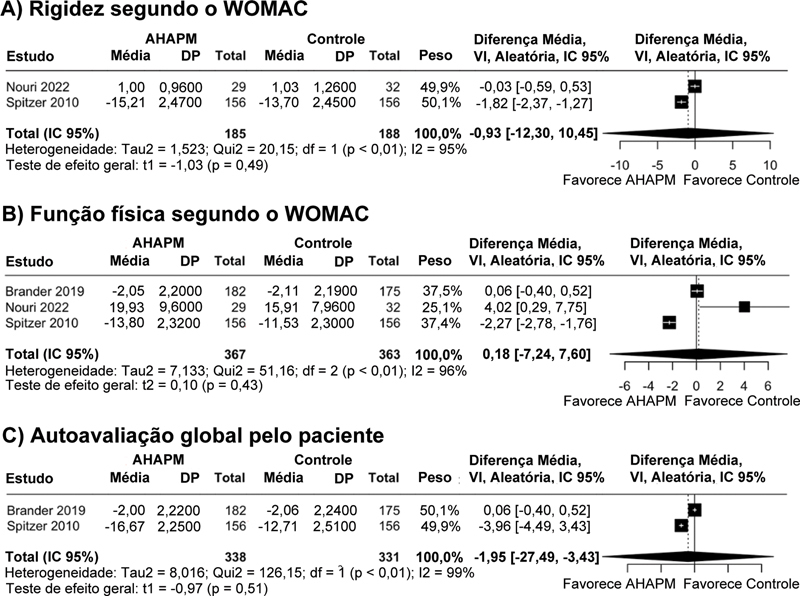
Gráficos de floresta comparando o ácido hialurônico de alto peso molecular (AHAPM) e outras terapias. ( **A** ) Rigidez segundo o WOMAC. ( **B** ) Função física segundo o WOMAC. ( **C** ) Autoavaliação global pelo paciente. DP, Desvio-padrão; df, graus de liberdade; IC, intervalo de confiança; VI, variância inversa.

Não houve diferenças significativas entre o tratamento com AHAPM e outras modalidades quanto à dor (DMP, −0,30 pontos; IC 95%, −1,60–0,99; *p* = 0,51; I ^2^ = 96%; Fig. 2A ), índice de Lequesne (DM, 1,30 pontos; IC 95%, −8,83 a 11,44; *p* = 0,35; I ^2^ = 12%; Fig. 2B ), WOMAC total (DM, −9,38 pontos; IC 95%, −51,33 a 32,56; *p* = 0,44; I ^2^ = 99%; Fig. 2C ), rigidez segundo o WOMAC (DM, −0,93 pontos; IC 95%, −12,30 a 10,45; *p* = 0,49; I ^2^ = 95%; Fig. 3A ), função física segundo o WOMAC (DM, −0,18 pontos; IC 95%, −7,24 a 7,60; *p* = 0,93; I ^2^ = 96%; Fig. 3B ) e autoavaliação global pelo paciente (DM, −1,95 pontos; IC 95%, −27,49 a 23,59; *p* = 0,51; I ^2^ = 99%; Fig. 3C ).

### Análise de sensibilidade

Na análise de sensibilidade *leave-one-out* , os resultados do desfecho de dor permaneceram estáveis. O gráfico da análise de sensibilidade *leave-one-out* é mostrado na **Figura Suplementar 1 (Fig. S1)** .

### Risco de Viés

**Fig. 4:**
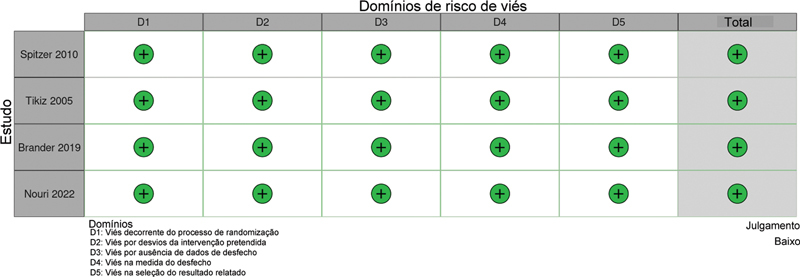
Avaliação crítica de estudos clínicos randomizados de acordo com a ferramenta Cochrane Collaboration para avaliação do risco de viés nessas pesquisas.

A Fig. 4 apresenta uma avaliação detalhada de cada ECR incluído na meta-análise, revelando um baixo risco geral de viés em todos os estudos.

## Discussão

Nesta revisão sistemática e meta-análise de 4 ECRs incluindo 823 pacientes com OA do quadril, comparamos o uso de AHAPM com intervenções controles. Não encontramos diferenças significativas entre os grupos em relação à dor, índice de Lequesne, pontuação total WOMAC, rigidez segundo o WOMAC, função física segundo o WOMAC ou autoavaliação global pelo paciente. A análise de sensibilidade confirmou a robustez do desfecho relacionado à dor.

A injeção intra-articular de AHAPM visa restaurar as propriedades do fluido sinovial, reduzindo o atrito e proporcionando alívio dos sintomas. 15 Embora bastante estudado e utilizado na OA do joelho, o uso de HA na OA do quadril requer mais pesquisas. Estudos sugerem que o peso molecular do HA está fortemente correlacionado à sua eficácia; assim, o AHAPM apresenta propriedades superiores, incluindo retenção intra-articular prolongada, efeitos condroprotetores robustos e melhor modulação dos processos inflamatórios. 16 17 Esses efeitos são bastante significativos ao considerar a meia-vida intra-articular relativamente curta do AHAPM, de cerca de 8,8 dias, o que contrasta com os benefícios clínicos contínuos observados. Essa discrepância é explicada pelos mecanismos biológicos do AHAPM, como a indução da síntese endógena de HA por fibroblastos sinoviais e a modulação de citocinas inflamatórias em médio e longo prazo. 17 Além disso, formulações como Sinovial HL (IBSA Farmaceutici Italia Srl), que combinam frações de alto e baixo peso molecular, demonstraram eficácia no tratamento da OA do quadril branda a moderada, reforçando o potencial terapêutico do AHAPM nessa doença. 15

A eficácia do AHAPM no tratamento da OA do quadril ainda é controversa. Richette et al. não observaram benefício clinicamente significativo com uma única injeção intra-articular de HA (Adant; peso molecular médio de 900.000 Da) em comparação ao placebo, sugerindo que uma única administração pode ser insuficiente para alcançar efeitos terapêuticos prolongados. 18 Em contrapartida, Spitzer et al. relataram que o Hylan G-F 20 produziu melhoras clinicamente significativas nos escores de dor e função, particularmente em pacientes com doença mais avançada, com manutenção dos efeitos por até 6 meses em comparação ao acetato de metilprednisolona. 1 Da mesma forma, Tikiz et al. 6 relataram que o Hylan G-F 20 e o AHBPM provocaram redução da dor e melhora funcional significativas até o 6° mês, sem diferenças significativas entre os 2 grupos, indicando eficácia comparável entre os diferentes pesos moleculares após a administração do HA em 3 doses semanais. Por outro lado, Brander et al. 5 não notaram diferenças significativas entre uma única injeção de 6 mL de Hylan G-F 20 e placebo em nenhum dos desfechos clínicos avaliados.

Recentemente, o PRP surgiu como uma terapia biológica promissora para a OA do quadril, visando a modulação da inflamação e o reparo do tecido articular. Nouri et al. 14 compararam o HA, o PRP e uma combinação de PRP e HA em pacientes com OA do quadril branda a moderada. Esses autores relataram que, embora todos os grupos tenham apresentado melhoras significativas nos escores WOMAC, VAS e Lequesne em até 6 meses, os grupos PRP e PRP + HA demonstraram resultados superiores em comparação ao grupo tratado apenas com HA, em especial nas atividades da vida diária e nos escores totais de Lequesne, com diferenças estatisticamente significativas observadas entre o segundo e o sexto mês.

Uma meta-análise de Gazendam et al. 19 demonstrou que as injeções intra-articulares de HA, combinadas ou não com PRP, não ofereceram nenhuma vantagem clinicamente significativa em relação ao placebo na OA do quadril; além disso, os autores não observaram nenhuma diferença significativa entre a AHBPM e a AHAPM. Diferentemente de nossos achados e da meta-análise de Gazendam et al. 19 sobre a OA do quadril, as injeções de HA têm demonstrado benefícios na OA do joelho, com algumas evidências sugerindo que o AHAPM pode apresentar melhor desempenho que o AHBPM nesse contexto. Os mecanismos subjacentes a essa aparente superioridade na OA do joelho ainda não foram esclarecidos, mas acredita-se que a maior viscosidade do AHAPM aumenta sua capacidade lubrificante. A anatomia esférica do quadril, em contraste com as superfícies articulares relativamente mais planas do joelho, pode diminuir a relevância dessa propriedade na OA do quadril; no entanto, essa hipótese carece de suporte empírico.

Este estudo tem diversas limitações. Primeiro, o pequeno número de estudos incluídos limita a generalização dos nossos resultados. Segundo, houve heterogeneidade estatística substancial entre as análises, o que pode afetar a robustez dos resultados. Portanto, ajustamos os ICs de 95% utilizando o método de Hartung-Knapp para aumentar a robustez da inferência de efeitos aleatórios. Terceiro, o uso de diferentes grupos controle entre os estudos incluídos introduz variabilidade, o que dificulta comparações diretas. Por fim, a impossibilidade de realização de análises de subgrupos de acordo com a gravidade da OA ou avaliações da influência de variáveis de confusão nos desfechos restringe a profundidade das nossas conclusões.

## Conclusão

Esta revisão sistemática e metanálise não observou diferenças significativas entre o AHAPM e outros tratamentos em relação ao alívio da dor e à recuperação funcional em pacientes com OA do quadril. No entanto, mais ECRs de alta qualidade são necessários para avalição do AHAPM no tratamento da OA do quadril.
